# Hashimoto's thyroiditis and acute chest syndrome revealing sickle cell anemia in a 32 years female patient

**DOI:** 10.11604/pamj.2015.21.142.6862

**Published:** 2015-06-22

**Authors:** Marielle Igala, Daniela Nsame, Jennie Dorothée Guelongo Okouango Ova, Siham Cherkaoui, Bouchra Oukkach, Asmae Quessar

**Affiliations:** 1 Hematology and Pediatric Oncology Service, Hospital of August 20 CHU Casablanca, Morocco; 2 Department of Endocrinology, University Hospital Casablanca, Morocco; 3 Laboratory of Hematology, CHU Casablanca, Morocco

**Keywords:** Hashimoto′s thyroitidis, sickle cell anemia, acute

## Abstract

Sickle cell anemia results from a single amino acid substitution in the gene encoding the β-globin subunit. Polymerization of deoxygenated sickle hemoglobin leads to decreased deformability of red blood cells. Hashimoto's thyroiditis is a common thyroid disease now recognized as an auto-immune thyroid disorder, it is usually thought to be haemolytic autoimmune anemia. We report the case of a 32 years old women admitted for chest pain and haemolysis anemia in which Hashimoto's thyroiditis and sickle cell anemia were found. In our observation the patient is a young woman whose examination did not show signs of goitre but the analysis of thyroid function tests performed before an auto-immune hemolytic anemia (confirmed by a high level of unconjugated bilirubin and a Coombs test positive for IgG) has found thyroid stimulating hormone (TSH) and positive thyroid antibody at rates in excess of 4.5 times their normal value. In the same period, as the hemolytic anemia, and before the atypical chest pain and anguish they generated in the patient, the search for hemoglobinopathies was made despite the absence of a family history of haematological disease or painful attacks in childhood. Patient electrophoresis's led to research similar cases in the family. The mother was the first to be analyzed with ultimately diagnosed with sickle cell trait have previously been ignored. This case would be a form with few symptoms because the patient does not describe painful crises in childhood or adolescence.

## Introduction

About 7% of the world's population's carriers for hemoglobin disorders (sickle cell anemia and thalassemia) [1]. Sickle cell anemia results from a single amino acid substitution in the gene encoding the β-globin subunit. Polymerization of deoxygenated sickle hemoglobin leads to decreased deformability of red blood cells [2]. It affects individuals of African, Mediterranean, and Asian descent and manifests as haemolysis and vaso-occlusion [3]. Adults with sickle cell disease (SCD) have the same symptoms as children. However, additional disease manifestations may present or worsen as patients age, including leg ulcers, sickle retinopathy, nephropathy, decreased bone density, thromboembolic complications, pulmonary hypertension, cardiac failure, transfusional iron overload, and vascular necrosis. Acute Chest Syndrome (ACS), often presents with clinical symptoms similar to pneumonia, is caused by vaso-occlusion in the pulmonary vasculature and is clinically described as the combination of hypoxia, fever, and a new infiltrate identified on chest X-ray. However, the clinical symptoms of hypoxia and fever often coincide with symptoms of vaso-occlusive episodes (especially in patients who receive narcotic medications) and may precede the radiographic changes, resulting in delayed diagnosis and treatment [3]. Fetal hemoglobin (HbF) is the major modifier of the clinical course of sickle cell anemia [4]. Hashimoto's thyroiditis is a common thyroid disease now recognized as an auto-immune thyroid disorder, characterized by goitre with lymphocytic infiltration and the presence of thyroid-specific auto-antibodies. It is usually thought to be haemolytic autoimmune anemia [5]. We report the case of a 32 years old women admitted for chest pain and haemolysis anemia in which Hashimoto's thyroiditis and sickle cell anemia were found.

## Patient and observation

**Figure 1:**
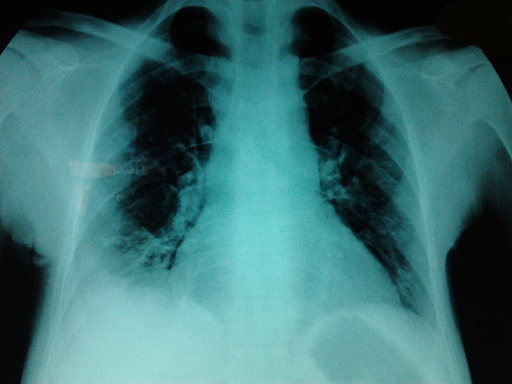
Right basal opacity on the chest X ray at diagnosis

**Figure 2:**
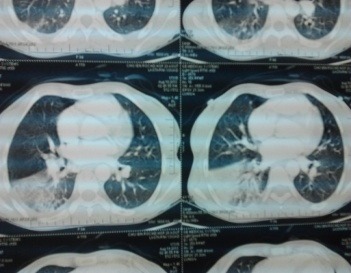
Chest angioscan at diagnosis showed a bilateral alveolar consolidation predominant on the on the right associated with minimal pericardial thickening and pleural effusion

**Figure 3:**
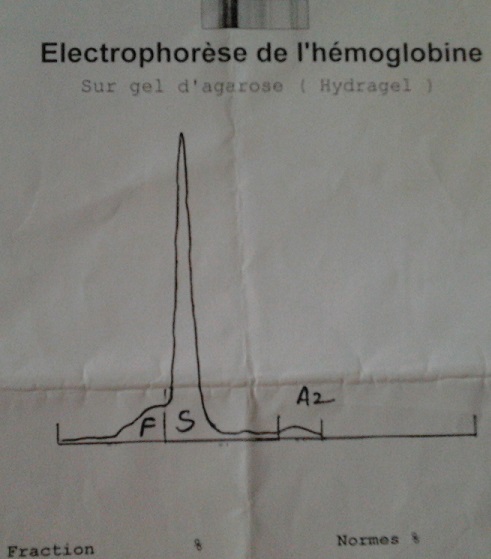
Electrophoresis of hemoglobin that revealed a migration of hemoglobin S and conclude to homozygous sickle cell anemia

A 32 years old female, with none known familial past medical history at this time, but a history of cholecystectomy 13 years ago, was initially addressed to hematology by the chest department for anemia. A few months after the first visit, she was admitted to hospital in an emergency setting for dyspnea and chest pain. Examination revealed acute respiratory distress syndrome, chest pain and jaundice associated with a fever of 38.5°C. The initial assessment found anemia of 6.9 g / dL, 105 fl VGM, leukocytes to 25.590 Giga/L with 18.510 Giga/L Neutrophils, 4.360 Giga/L Lymphocytis, 2.630 Giga/L Monocytis, 0.9 Giga/L Basophile and 240 Giga/L Platelets. The coagulation tests were normal, the total bilirubin 95mg/L (Normal range: 1-12 mg/L) unconjugated bilirubin 83mg/L, conjugated bilirubin 12 mg/L (Figure 1), echocardiography found a minimal cardiac effusion with an ejection fraction at 58% and pulmonary arterial pressure at 31mmHg, normal abdominal ultrasound, venous Doppler of lower limbs was normal, the chest CT angio-scan did not find a pulmonary embolism but bilateral alveolar consolidation predominant on the right associated with minimal pericardial thickening and pleural effusion (Figure 2). The bone marrow biopsy was normal. The patient started steroid treatment but had stopped it by invoking bone pain. The biological exams were completed by immunologics tests: Antinuclear Antibody (normal), anti DNA native antibody (normal), T3 free hormone 0.7ng/ml (0.69-2.02ng/ml / T4 free hormone 4.9µg/dl (4.8-11.6µg/dl) and thyroid-stimulating hormone (TSH) 27.6 mUI/L (4.5 X Normal range: 0.3-6.2 mUI/L) Antibody Anti-Thyroperoxydase 151.1 UI/ml (4.5 x Normal range 0-34 UI / ml). The hemoglobin electrophoresis found a homozygous sickle cell disease (HbS: 78.5%, and 18.1% HbF, HbA2 3.4%) (Figure 3). These results led us to further questioning of the patient in search of a family history of sickle cell disease and painful episodes in childhood or adolescence. The patient did not report any painful episodes in childhood but had a maternal aunt followed in hematology for sickle cell disease. The hemoglobin electrophoresis realised for her mother concluded a heterozygous sickle cell anemia (HbS 35, 2%, HbA 60.8%, HbA2 3%, HbF 1%).

## Discussion

In our observation the patient is a young woman whose examination did not show signs of goitre but the analysis of thyroid function tests performed before an auto-immune hemolytic anemia (confirmed by a high level of unconjugated bilirubin and a Coombs test positive for IgG) has found thyroid stimulating hormone (TSH) and positive thyroid antibody at rates in excess of 4.5 times their normal value. In definitive the diagnosis was hemolytic anemia due to Hashimoto's thyroitidis. The incidence of Hashimoto's thyroiditis is 0.3-1.5 cases per 1000 population per year. The prevalence of positive antibody tests in women is greater than 10% and of clinical disease at least 2%. Men have one tenth of this prevalence. Clinical diagnosis is based on the presence of diffuse goiter (or various clinical courses including euthyroidism with goiter, subclinical hypothyroidism with goiter, hypothyroidism, adolescent goiter), anti-thyroid peroxidase Ac, anti-Tg Ac or lymphocytic infiltration on cytological examination [5]. In the same period, as the hemolytic anemia, and before the atypical chest pain and anguish they generated in the patient, the search for hemoglobinopathies was made despite the absence of a family history of haematological disease or painful attacks in childhood. Patient electrophoresis's led to research similar cases in the family. The mother was the first to be analyzed with ultimately diagnosed with sickle cell trait have previously been ignored. The patient reported that one of her maternal aunts was followed in hematology for sickle cell disease. Sickle cell anemia symptoms start at 6 months of age (as fetal hemoglobin dissipates) with dactylitis (painful swelling of the hands or feet), anemia, mild jaundice, or an enlarged spleen. Adults have the same symptoms as children. Acute pain episodes have a peak at age 20-29 years. Adult patients who report more than three pain crises per year have a predicted decreased survival [3]. In our patient sickle cell anemia was diagnosed at an unusual age. The history of cholecystectomy should have alerted us to a previous chronic hemolysis. This case would be a form with few symptoms because the patient does not describe painful crises in childhood or adolescence. Many genetic factors are involved in the regulation of the intensity of the clinical features. One of the best known and most studied of these is the Fetal hemoglobin (Hb F). High HbF is strongly associated with a reduced rate of acute painful episodes, fewer leg ulcers, and longevity [6]. Fetal hemoglobin inhibits the polymerization of hemoglobin S and thereby reduces the complications of the disease. This is also the protective effect of this molecule which is sought when adding the hydroxy urea in the management of sickle cell patients. Lower HbF levels correlate with overall more severe disease manifestations [7]. Fetal hemoglobin concentration higher than 15% prevents sickle globin polymerization. The cut-off for defining lower risk of severe complications has been estimated at 20% [8]. The estimated value at 18% of HbF for this patient seems to play a role in attenuating the intensity of the clinical manifestations in spite of the cut-off of 20%.

Acute Chest Syndrome is an acute lung injury syndrome that occurs frequently in patients with SCD. This lung injury syndrome has been defined in clinical research studies as a new pulmonary infiltrate on chest X-ray consistent with alveolar consolidation but not atelectasis, involving at least one complete lung segment. The radiographic abnormality is usually accompanied by one or more new signs or symptoms, including chest pain, fever, tachypnea, wheezing, cough, or hypoxemia. The etiology of ACS is multifactorial. The three primary studied mechanisms include pneumonia or systemic infection, fat embolism, and direct pulmonary infarction from HbS-containing erythrocytes [9]. In our case, a pulmonary embolus was the first suggested diagnosis behind the chest symptoms but it was difficult to establish. Indeed pulmonary emboli may contribute to pulmonary symptoms associated with acute chest syndrome and pulmonary hypertension and while identified in up to 50% of autopsies of sickle cell disease cases, but only 5% are detected clinically, in part because they are difficult to distinguish from in situ thrombosis [10]. Chest pain and all other signs that accompanied it and that motivated the hospitalization of the patient may respond to a real acute chest syndrome in sickle cell disease. This is supported by the radiological assessment evidence. The assessment of hemolysis in our patient led us to not omit the possibility of sickle cell disease depended on the age and positive Coombs test that would have explained the hemolysis but not the chest pain. The thyroid has been systematically evaluated as would the diagnostic approach to hemolysis. Sickle cell patients can develop any kind of pathology. Thus the diagnosis of thyroiditis should not be an exception. A case of parathyroiditis has also been described in a 17 year old boy with sickle cell anemia [11].

## Conclusion

This unusual event of discovery of sickle cell disease in an adult who had no previous antecedents other than a cholecystectomy is witness of hyperhemolysis which illustrates the probability of a genetic involvement in the onset of disease manifestations. Otherwise independent of its hemolytic character all other causes of hemolysis should not be forgotten in a sickle cell patient.
